# Next-Generation Capabilities in Trusted Research Environments: Interview Study

**DOI:** 10.2196/33720

**Published:** 2022-09-20

**Authors:** Sanaz Kavianpour, James Sutherland, Esma Mansouri-Benssassi, Natalie Coull, Emily Jefferson

**Affiliations:** 1 School of Design and Informatics Abertay University Dundee United Kingdom; 2 Health Informatics Centre University of Dundee Dundee United Kingdom

**Keywords:** data safe haven, health data analysis, trusted research environment, TRE

## Abstract

**Background:**

A Trusted Research Environment (TRE; also known as a Safe Haven) is an environment supported by trained staff and agreed processes (principles and standards), providing access to data for research while protecting patient confidentiality. Accessing sensitive data without compromising the privacy and security of the data is a complex process.

**Objective:**

This paper presents the security measures, administrative procedures, and technical approaches adopted by TREs.

**Methods:**

We contacted 73 TRE operators, 22 (30%) of whom, in the United Kingdom and internationally, agreed to be interviewed remotely under a nondisclosure agreement and to complete a questionnaire about their TRE.

**Results:**

We observed many similar processes and standards that TREs follow to adhere to the Seven Safes principles. The security processes and TRE capabilities for supporting observational studies using classical statistical methods were mature, and the requirements were well understood. However, we identified limitations in the security measures and capabilities of TREs to support “next-generation” requirements such as wide ranges of data types, ability to develop artificial intelligence algorithms and software within the environment, handling of big data, and timely import and export of data.

**Conclusions:**

We found a lack of software or other automation tools to support the community and limited knowledge of how to meet the next-generation requirements from the research community. Disclosure control for exporting artificial intelligence algorithms and software was found to be particularly challenging, and there is a clear need for additional controls to support this capability within TREs.

## Introduction

A Trusted Research Environment (TRE), sometimes also known as a Data Safe Haven, Virtual Research Data Center, or Virtual Data Enclave, is a secure environment designed for approved and named researchers to access sensitive data, where access to specific data sets is provided to approved research projects. To protect the confidentiality and privacy of the data, TRE providers and researchers using the environments generally follow a set of TRE principles. Such principles have developed over time, for example, the Scottish Safe Haven Charter and the Health Data Research Alliance Trusted Research Environment Green Paper [1-3].

The objective of a TRE is to provide safe and trustworthy access to data for research. Controls are generally applied to both the import and export of data to protect the privacy of data subjects and the integrity of the environment itself. For example, a researcher may be given access to a data table listing the age, medication taken, and hospital admission information on each of 5000 people. They will be allowed to identify and publish aggregate statistical observations that people aged >60 years who are taking a particular drug were x% less likely to be admitted to the hospital with heart problems, but the details of each individual will never leave the TRE.

Within the secure environment, researchers can analyze these data using a set of advanced analytics tools, for example, R and SPSS. Some TREs also offer the researcher the capability to program within the environment, support the development of new artificial intelligence (AI), and apply natural language processing for the analysis of unstructured text.

Many TREs have been developed to host health data. For example, in the United Kingdom, several TREs are established to host health records from the National Health Service (NHS), the publicly funded health care system of the United Kingdom [4-8]. A similar model has since been adopted to provide secure access to many other non–health-related data sets [9-11]. Many TREs now regularly host both health-related and non–health-related data. Providing researchers access to sensitive data sources without compromising the privacy and security of the data is a complex process. Historically, TREs have mainly supported observational studies on text-based structured data using standard statistical packages. There is a growing requirement from the research community (academic and industrial) for TREs to provide additional capability beyond simple support for observational data statistical analysis, but without compromising the security or privacy of the data. In this paper, we term these requirements as next-generation TRE capabilities. These include the following:

1. Support for big, nonstructured data (such as genomic and imaging data, which can be several terabytes in size)
2. Ability to parallelize computational jobs to either a high-performance computing cluster or a graphics processing unit (GPU) farm
3. Support for software development within the TRE
4. Freedom to install software packages of researcher choice
5. Ability to export software and AI algorithms from the environment
6. Ability to connect to certain internet locations, for example, code repositories (GitHub)

This study aims to understand the state of the art in supporting next-generation TRE capabilities; existing technical security measures that have been adopted and how widely; and limitations in existing controls and processes, where active studies are required to develop novel methods.

## Methods

### Participant Recruitment

A total of 73 individuals from 42 different organizations (n=22, 52% in the United Kingdom and n=20, 48% overseas) were invited to participate, but response rates were substantially higher in the UK cohort (20/73, 27% responses vs 7/73, 10% responses), perhaps owing to greater familiarity. A few respondents either declined or were unable to participate for practical reasons, and 3% (2/73) of them felt that their situation was already covered by other participants from related institutions, so they were not counted separately.

The findings are based on interviews conducted with 84% (16/19) of TRE providers in the United Kingdom and 16% (3/19) of TRE providers across Canada, Australia, and Europe. Each interview took approximately 2 hours, using a set of questions that were designed to cover TRE controls and next-generation capabilities (Multimedia Appendix 1).

Building upon the Five Safes model [12], the recent HDR UK Green Paper [2] describes seven “Safes”: safe people, safe projects, safe setting, safe computing (an extension of safe setting), safe data, safe outputs, and safe return (extending the TRE definition). This study focuses on a subset of the controls that will support next-generation TRE capabilities (data, outputs, settings, computing, and people). Textbox 1 provides a summary of the topics that were discussed with the participants during the interview, under each of our subset of the 7 Safes.

The interview participants were recruited mainly from the technical TRE infrastructure teams. A nondisclosure agreement was signed by the project parties, to provide assurance for any participant who chooses to disclose information of confidential or proprietary nature. We have anonymized the responses in this paper and grouped them under different Safes. There were some differences in some controls and measures from TREs from different countries, but we could not directly highlight these as they may have affected the anonymity of participants.

Where relevant, we also present our analysis of the TRE limitations identified and recommendations that we believe can help improve TREs.

**Safes and discussion points.**

**Safe data**

- Tools and techniques used to manage and reduce the potential risk of reidentification by applying disclosure control to all data imported to the Trusted Research Environment (TRE)

**Safe outputs**

- Types of data that can be exported from the TRE
- Future plans to enable export of additional types of data
- The process used for checking disclosure control on data to be exported, including frequency and restrictions
- Software and any manual checks used for disclosure control

**Safe settings**

- Standard build of the TRE (including computing power, operating system, and software)
- Maximum computing power offered to TRE “power” users
- Security measures used to mitigate the risk of unauthorized access, data loss, and misuse
- Rules regarding the import of data or code (including libraries) into the environment
- Support for federated queries of data from external sources

**Safe computing**

- Use of private (dedicated hardware) or public cloud (paying for capacity from a service such as Amazon Web Services or Azure)

**Safe people**

- The controls put on the people who use the TRE
- Whether access to the environment must be via a recognized “trusted” organization

### Ethics Approval

The work presented in this paper was considered by Abertay University Ethics Committee on May 28, 2020, and granted full approval, with reference number EMS3012.

## Results

### Safe Data

#### Overview

The principles of safe data relate to the data allowed to be imported into the TRE. Good practice indicates that such data should be of high quality and pseudonymized or anonymized [2]. Researchers accessing the TRE should only be able to access the data necessary for their research project; the work of protecting data begins by applying disclosure control to control and assess data provided to the researchers within the TRE, before a second pass at the export stage regulates the data those researchers are allowed to disclose publicly within their research output.

Many TREs provide a service to link and anonymize data from different data sources. Such research projects often require data governance approval before researchers can access the data. TREs often require researchers to sign data user agreements, which reinforce the rules and consequences of violations. During the interviews, participants were asked to discuss the existing tools used to support safe data and their views on future solutions. These are discussed in the following sections.

#### Breaches or Near-Miss Incidents

Each participating TRE has processes in place for incident response, including reporting to the appropriate authorities when required. None of them reported any actual reportable incidents. In total, 9% (2/22) of the participants acknowledged that there can be lapses of procedure (these are different from data breaches and do not need to be reported to the Information Commissioner’s Office), where a researcher may request to export data that are not permitted, perhaps owing to researcher error. In these cases, the incident will be addressed with a process for review, formal warning, and retraining, if necessary. One participant described an incident in which a field that normally contains facility or hospital names was provided to a researcher, and some records included private addresses in this field. Then, these data were retracted and resupplied with a placeholder for private addresses. In this example, the data were not released outside the TRE, but researchers saw the potentially identifiable data within the TRE environment.

#### Tools and Techniques Used to Manage the Risks of Reidentification

##### Overview

Most participants (16/22, 73%) do not rely on special purpose disclosure control tools, but on their analysts’ knowledge and communication about the purpose of the project and the nature of the data. Ultimately, TREs exist with the aim of providing “safe data” sufficient for the project’s needs. Generally, patient IDs are replaced with either TRE-specific or project-specific identifiers; where possible, other identifying information is redacted or reduced in resolution, for example, replacing a date of birth with a year or month of birth. The combination of data across projects is not allowed and is made impractical through the use of project-specific pseudonyms in place of original identifiers.

A total of 18% (4/22) of the participants explicitly measure disclosure risks at the import stage for each project; however, all participants (22/22, 100%) acknowledged that disclosure risk is an important factor when evaluating a new project application. As part of this process, a TRE has all data scrutinized by an external organization, using the k-value (the minimum number of individuals sharing any combination of identifying characteristics) to quantify the reidentification risk for each data set [13]. For example, a data set classifying patients by age and gender, with a minimum of 4 patients in each category, has k=4. Grouping patients into larger age bands will increase the k-value, thus reducing risk at the expense of reduced data resolution. TREs apply a similar measure as part of the export process, usually requiring a minimum of 5 to 10 individuals in any output grouping to ensure that no individual can be reidentified, as detailed in section 3.2.

A total of 18% (4/22) of the participants reported using tools to support checks during the import and export processes. Each of them used a different tool, as follows:

1. sdcMicro (as discussed in section 2.6) is a free R-based open-source package that assesses the risk of a data set containing identifiable data using various risk estimation methods [14].
2. Privitar’s Data Privacy Platform product applies user-defined policies for filtering and transforming data, including adding random “noise” to numerical values in configurable ways to reduce identifiability [15].
3. Custodix Anonymisation Tool Services deidentification platform [16] provides both assessment and deidentifying transformation of various types of data (eg, comma separated variables and Digital Imaging and Communications in Medicine).
4. Privacy Analytics Risk Assessment Tool [17] is used by a TRE to assess risk, redact, and deidentify source data and to assess export requests.

##### Recommendations

There is a need for affordable tools that can be used in TREs to support deidentification of data and assess the risk of reidentification; in particular, building on the existing use of the free sdcMicro toolkit should be considered. Data identifiability is not binary [18], and data can be identified indirectly by combining attributes, which is known as a triangulation attack [19].

### Safe Outputs

#### Overview

Participants were asked which data types can be exported from their TRE and what controls are placed on the export of data. These checks are more extensive than those for imported data, adding checks for deliberate attempts to hide data, for example, as white text in the document or embedded in Stata code and generic checks on the actual data going further than the input checks—usually an absolute prohibition on data regarding any individual (“row-level”).

Generally, the researcher explicitly requests the export of specific files, which are then reviewed by TRE staff, and in some cases, other relevant external parties (eg, data owners) before the permission to export is granted. All the participants have instructions that document the manual checks required. The team determines the extent of checks required at the data or project level based on the sensitivity of the data. For example, openly available, public data sets will not typically require an independent referee, but clinical data may require more consideration. To obtain highly sensitive data, multiple members of the review team may be required to check each file.

The checks needed varied between TREs and sometimes between projects, depending on the nature of the data, including how sensitive it is and whether it was consented or not, with release criteria being agreed upon between data owners and TRE operators, sometimes consulting with research teams for specific situations and then enforced by TRE staff. Many TREs have developed a rule-based framework to categorize projects and data into specific types. For example, open public data can be exported with minimal checks, whereas for clinical data, only aggregate-level summary data can be exported. Others used a simpler *one-size-fits-all* approach.

#### Export of Individual-Level Data

Generally, export of row or individual-level data is permitted only for projects where the data are already openly available in the public domain or where specific consent had been provided by the study participants to collect and share the data. In the latter case, respondents indicated that the data controller was most likely to be the principal investigator of the project, and they will typically use the TRE to securely manage access to their data by different researchers involved in the project, but will choose not to place restrictions on data export. Otherwise, only aggregate statistics can be exported.

#### Export of Aggregate-Level Statistical Analysis

Most TREs only allow export of aggregate-level statistical analysis. For example, in clinical data projects where the data controller is an NHS board or trust, researchers are not permitted to export any data related to specific individuals (even if pseudonymized).

All participants (22/22, 100%) indicated that their TREs allow the export of aggregate-level data as graphs or tables, with a minimum number of data points in any table cell or graphical output to reduce the probability of reidentification of data from small sample sizes (“small cell risk”). Of the 22 participants, 17 (77%) participants have set policies in place: 32% (7/22) of the participants reported using a minimum of 5 individuals in a cell, with 9% (2/22) of the participants using 10 and 36% (8/22) varying the limit depending on the context of the study and the nature of the underlying data.

All participants (22/22, 100%) acknowledged that there was a potential risk of reidentification if sufficient data points are exported from the TRE, known as jigsaw identification or triangulation [20]. Although there is a clear need for researchers to export aggregate-level statistical analysis, the mitigations used for these risks vary across different data sets [3]. Software tools can be used to estimate the probability of reidentification, as discussed in section 2.2.

Introducing new types of data as export options brings new risks, particularly AI models and software, where statistical analysts will be unfamiliar with the nature of such files and manual inspection is ineffective, difficult, and time-consuming, thus introducing new security risks. Data can be intentionally concealed within such files and recovered by a third party from an exported model created innocently through means such as membership inference attacks [21] or, with some internal knowledge or collusion with a researcher, inversion attacks to deduce additional information about the training data [22].

In the following section, we discuss the different data types that can be exported from the participants’ TREs and how this is managed.

#### Export of AI Algorithms, Software, and Scripts

##### Overview

Participants were asked about policies regarding the export of software and AI models developed within their TREs. Of the 22 participants, 5 (23%) allow export of AI models, 8 (36%) specifically prohibit this, and 5 (23%) are prepared to consider it in the future.

Some participants also plan to support the export of R and Stata scripts in the future, if they have established a suitable process for reviewing.

A total of 74% (14/19) of the TREs permit the export of software source code developed within the TRE. None of them (0/19, 0%) have been asked to allow compiled executables, 11% (2/19) are prepared to consider this with safeguards. However, it should also be entirely avoidable by developing the source code outside the TRE and then deploying it into the TRE for testing [23].

##### Limitations

Many participants (9/22, 41%) indicated that checking algorithms, software, and scripts is very challenging because a malicious individual can “hide” individual-level data within the files. For example, the weights of an AI algorithm are a set of numbers and sensitive data can be embedded in them. This is very difficult to detect, particularly if a malicious user disguises the data. It is also possible to include individual-level data inadvertently, for example, if the AI algorithm is overtrained, the weights correspond to the data underneath, or if an R script incorporates the underpinning data. Checking a substantial software project manually is unrealistic.

##### Recommendations

Developing AI models in TREs without compromising patient privacy requires tools such as those proposed by Nicolae et al [24] and Liu et al [25] to quantify their risk and vulnerability to attacks (eg, membership inference attacks [26,27], deanonymization attacks [28], reconstruction attacks [29], model extraction attacks [30,31], and model inversion attacks [23,32]) and consider integrating privacy mechanisms in the model development to counter these attacks [33]. Best practice guidelines can also help users to design robust and safe algorithms, including through auditable and explainable AI [34]. Software tools to check for nonmalicious export by comparing individual data within the TRE with those in the export files is a possibility, but such tools are not currently routinely used by any of the TREs. Barriers to their use by TREs include the attack-specific nature of such tools and their high price. For software development (as opposed to AI models, where the training data are essential input for the end product), exporting the software from the TRE can be avoided entirely by developing it outside.

#### Automation of Data Export Checks

##### Overview

Although software can theoretically be used to facilitate the data export process, none of the participants (0/22, 0%) believed that software can currently replace the role of humans for checking export files. A participant questioned whether it would ever be feasible to fully automate all aspects of the process, largely owing to concerns about trusting the software to perform all the necessary checks without human oversight. Some participants (8/22, 36%) felt that the software available is currently not sufficiently mature to manage all the risks and humans are better at the task; however, they indicated that they would be willing to incorporate software of this nature into the process in the future as the technology evolves.

Of the 22 participants, 2 (9%) participants reported using automated tools for export checks. As noted in section 2.2, one participant used the proprietary Privacy Analytics Risk Assessment Tool product and another participant used a simple in-house tool to detect the project-specific identifiers they use, but the main disclosure checks are manual. Currently, the use of automated tools seems to be more prevalent for import checks than for export checks.

##### Limitations

Manual checks are time-consuming and error-prone, with a risk of missing concealed data (steganographic, white-on-text, and undo buffers) and delays in data release. Although the participants acknowledged that the current data center process can be enhanced with automated tools, there are significant concerns with relying solely on technology to check export requests, based on the potential ramifications for any unapproved data to accidentally leave the environment and the challenges with checking algorithms. Proprietary tools are expensive, and TREs try to keep the costs low for academic research.

##### Recommendations

A hybrid model with automated checks can facilitate and accelerate export and reduce the risk of reidentification, checking more thoroughly for inadvertent and malicious inclusion of data. The tools mentioned in section 2.2 may also be useful in this role. Best practice guidance regarding methods to reduce the opportunities for malicious data exfiltration can also help. Although governance (section 6) can help to ensure that researchers are trustworthy, malicious attempts to hide data should be considered, for example, in the event of stolen researcher credentials.

#### Frequency of Data Exports

##### Overview

All participants (22/22, 100%) reported that researchers can request data to be exported from the TRE at any time; however, the frequency of requests varied significantly per project, with some requiring daily exports and others exporting only at the end of a project.

Participants were keen to explore how the review process of data export can be improved and automated to decrease the review team’s workload. However, there was concern that more frequent exports increased the risk of data leakage. For example, 2 consecutive releases featuring a subgroup of 26 and 27 patients, respectively, will each be acceptable in isolation, but comparing the 2 subgroups discloses additional information about the additional patient in the second release.

##### Limitations

As the manual export checking process uses significant staff time, some TREs apply limits on the number of exports or charge projects more for frequent use. A participant explained that the volume of export requests allowed is related to the cost model of the tenancy. For example, a TRE allowed only 2 releases for MSc or BSc projects.

##### Recommendations

Owing to the different types of data used across the different TREs and the different types of projects it is evident that there is no “one-size-fits-all” solution, but rather a solution needs to be sufficiently flexible to facilitate these differences between projects, data sets, and TREs. Automation can help to address these resource concerns and increase the speed and frequency at which researchers are able to export data. Although human checks are useful, the process has limitations and the risk of human error.

#### Potential Gaps in Export Checks

##### Overview

Participants were asked whether they perceived any gaps in the export process and how they thought it can be improved. Of the 22 participants, 15 (68%) were not aware of any gaps or security concerns. The following concerns were raised by the other participants:

1. Researchers can be creative in finding a way to remove data, for example, by using screen capture to exfiltrate data, which will be difficult to detect.
2. Manual checks have the potential for human error.
3. Owing to the variety of data types that will be requested for export, it was difficult to find software that had the functionality to check all the file types. This variety also makes it challenging to bring together a review team with knowledge of where data may be accidentally or deliberately hidden, particularly for novel data types. None of the TREs were aware of any existing software tools that can be used for checking algorithmic data export requests.
4. Deficiencies in the audit trail make it impossible to see what the researchers have done in the TRE, because, sometimes, studies may have deviated from the original goal, and this was difficult to detect.

A participant mentioned that the manual process can be greatly enhanced by the following:

1. Effective training
2. Ensuring that staff rigorously check outputs
3. Applying the principles of appropriate frameworks, such as the Seven Safes, and nationally recognized “best practice” (eg, the Canadian essential requirements for operating data trusts [35])
4. Having a collaborative relationship with researchers throughout their project to mitigate and prevent malicious behavior

##### Recommendation

Typically, the 2 different models for output checking are principle-based and rule-based models [36,37]. The principle-based model better ensures confidentiality protection and utility of outputs from a statistical perspective, at the expense of being hard to standardize, automate, and verify.

### Safe Settings

#### Overview

The safe setting controls cover the infrastructure and associated security measures that should be adopted by TREs. These controls specify that computing power and operating systems should enable a safe setting to sustain both economical scalability of compute for analysis (eg, images and genomics) and integral data security. Safe setting controls describe the best practices of policies, techniques, and security measures and strategies that are required when sharing data for analysis.

#### Computing Power and Operating System Offered to a “Standard” User

##### Overview

Generally, TREs take the form of a virtual desktop infrastructure—each user receives remote access to a desktop environment with access to their project’s data and appropriate software to analyze that data. Most TREs provide each user with their own virtual machine (VM), with fixed resources (particularly memory and processing) isolated from other projects and users, whereas a few TREs share a multiuser system more directly (known as “session-based” virtual desktop infrastructure), allowing a user to exploit the full hardware capacity of the host system when needed, at the expense of reduced isolation between users and projects.

Alternative approaches also exist; for example, the OpenSAFELY platform [38] provides indirect access to patient data. Rather than manipulating the data interactively, researchers develop analysis scripts against synthetic dummy data and then submit those scripts for remote execution. None of the groups that provide access via such alternative approaches agreed to participate in this study; therefore, they were not included in the analysis.

Most respondents (18/20, 82%) indicated that standard templates were usually used for the TRE and the computing power offered for a project will depend on the number of users who need to access it. Of the 22 participants, 1 (5%) creates custom configurations for every project, 2 (9%) have no flexibility available, and 12 (55%) reported that their TREs can scale-up depending on the researcher’s requirements. Heavy compute usage will have high costs associated with it, which can be a barrier for many research projects. A participant mentioned that the maximum computing power configurations depend on each individual project’s budget constraints. Table 1 lists the different computer power available across the TREs (some of the participants were not able to answer this question and some use the public cloud, so that resources are effectively limited only by budget).

A total of 53% (10/19) of the TREs reported that Windows (including Windows 10, Windows Server 2012, Windows Server 2019) was the standard build operating system. Of the 19 TREs, 4 (21%) responded that they can provide both Windows and Linux based on the researcher’s request. In a TRE, Ubuntu was the only standard build. From the participants’ responses, it was evident that there was great variety in the specifications available, with some having multiple orders of magnitude more capacity than others.

**Table 1:** Available computing power.

Processing power and RAM			Storage space		Allocation
1 CPU^a^	8 GB	5 TB		VM^b^	
2 CPUs	8 GB	250 GB (fast scratch)		VM	
4 CPUs	16 GB	—^c^		VM	
1.5 cores	18 GB	1 TB		VM	
4 to 8 cores	32 to 64 GB	60 to 80 GB		VM	
4 to 64 cores	—	8 GB to 2 TB		Host	
16 cores	—	96 GB		Host	
**Dual Xeon processor**					
	—	120 GB		VM	
	~2000 cores	—		Host	
**GPU** ^d^ **cluster**					
	—	200 TB		Host	
	4 TB	32 TB		Host	

^a^CPU: central processing unit.

^b^VM: virtual machine.

^c^Information not available or no predetermined value.

^d^GPU: graphics processing unit.

##### Limitations

Although most current researcher needs are met by existing TREs, it is clear from Table 1 that some TREs can find it challenging to support processor-intensive projects. Furthermore, most, but not all, of the TREs provide each project its own isolated VM, which can have implications for isolation and pose an increased security risk if malicious code was able to run and potentially access other projects and their data within a shared system as opposed to a project-specific VM.

##### Recommendations

TREs should consider the scalability of their infrastructure to support resource-intensive projects in the future. Use of public cloud infrastructure enables much greater flexibility, for a price, and incorporates robust isolation between VMs as standard.

#### Data Security Measures Used in TREs to Mitigate the Risk of Unauthorized Access, Data Loss, and Misuse by Researcher

##### Unauthorized Access

The participants discussed different measures that were implemented to help prevent unauthorized access. Different controls were implemented across the participating TREs, depending on the underlying infrastructure. We present a full list of the controls that were discussed during the interview; however, not all the TREs implemented the full list of controls described in this paper:

1. Best practice password policy (which will include lockout after 2 or 3 incorrect attempts)
2. Access controls
3. Access to TRE only permitted via white-listed IP addresses
4. Fully automated account management
5. Sensitive projects may have restrictions on the location of the researcher (in its strictest form, this can include permitting access only from a specific room [on campus] and via managed devices [restricted machines], or more generally, permitting IP addresses only from particular countries)
6. Manage file access
7. Active Directory hierarchical privileges
8. Session recording
9. Monitoring or audit system, such as IBM Guardium, SIEM, and Splunk
10. Multifactor authentication
11. Network segmentation
12. Compartmentalization to limit access to information to entities on a need-to-know basis to perform certain tasks, to protect information vendor firewalls (3 different vendors)
13. Patch management
14. Biannual pen testing

##### Data Loss

In the TREs, internet access is blocked, and users have limited access rights. The remote access is designed to prevent moving data in and out of the environment, except via the official channels, with appropriate controls in place: virtual hardware ports and copying data to the client system’s clipboard are disabled. Some TREs also take steps to impede pasting; however, this is not reliably achievable (the direct paste shortcuts can be disabled, but it is trivial for a knowledgeable user to bypass this with a single command on client systems). Measures are also established to detect attempts to export data via other routes. Antimalware or antiransomware software and data loss prevention software are used.

##### Misuse by Researcher

The main countermeasure to misuse by researchers is training. Generally, this reinforces key principles to ensure that researchers understood their responsibilities and what activities are permitted or not permitted within the environment. Other significant mitigation strategies are checking the outputs and reviewing the project’s scope. In 11% (2/19) of the TREs, researchers must be accredited by a particular organization before they are granted access to the environment (this accreditation requires the researchers to prove that they have appropriate qualifications and experience). Furthermore, researchers must sign an investigator’s declaration stating that they will not misuse the environment, and the line managers and organizations will be held accountable if a user attempts to do anything malicious.

A TRE uses session recordings to help detect misuse. In this TRE, researchers’ behavior such as keystrokes can be monitored. Another TRE uses a monitoring program from Darktrace to detect a user running a tool on their laptop to take screenshots [39]. Other 2 TREs have a full audit log from log-on to log-off, and another TRE plans to log activities to enable reconstruction in the event of a breach.

Many other controls were discussed, which included the following:

1. Researchers are not granted admin access in the TRE.
2. Researchers have access only to their own project’s TRE storage.
3. Printing, mapping drives, and accessing external drives are not allowed.
4. Command prompt access is disabled.
5. ISO27001 policy rules via a cloud security posture management system.

##### Recommendations

The previously mentioned examples of current practices to detect and prevent instances of unauthorized access, data loss, and researcher misuse should be considered by all TREs to further improve security, where appropriate for the specific TRE infrastructure. Furthermore, TREs must have a legal agreement constraining access and use as their data security measure to mitigate the risk of misuse by the researcher. Programs to monitor and record researchers’ behavior are also useful to reduce misuse.

#### Importing of Data or Code

##### Overview

Participants were asked if they allow researchers to import data or code (including libraries) into the environment and, if so, what security measures (eg, software) are used to support this process:

1. A total of 63% (12/19) of the TREs allow the import of both code and data.
2. A total of 16% (3/19) of the TREs allow code (with some restrictions), but not external data sets.
3. A total of 11% (2/19) of the TREs allow data, but not code.
4. A total of 5% (1/19) of the TREs allow neither.

The import of data or code is subject to gatekeeper approval with a check that the import does not contain hidden data and that the code does not pose a threat to the security of the TRE. This gatekeeper approval process varies between TREs, but typically involves manual checks. In addition to scrutinizing the security risk posed by the data or code, this process can also involve checking the file size, file type, magic numbers, and known suffixes. In general, this process is supported by virus scans, static code analysis tools, and sample code execution in a sandboxed environment.

Some participants discussed the important role of “trust,” and how training the researchers and trusting that they have no malicious intent is sufficient, based on the low risk of potential damage from malicious code and subject to low sensitivity of the data (refer to the Safe People section for more detail). Finally, a participant mentioned the role of monitoring to detect any malicious behavior, so that inappropriate or malfunctioning software can be identified.

##### Limitations

There was substantial reliance on manual checks to support this process. Furthermore, participants had clear concerns about the security implications of importing malicious code. The main concerns regarding the process of supporting code or data egress were highlighted as follows:

1. Ensuring that the AI algorithms or software imported into the environment do not include sensitive data.
2. It can be extremely time-consuming for the TRE staff or researchers to manually import code after each small change.

##### Recommendations

Some of these security concerns can be mitigated by isolating each project within the TRE to minimize potential damage and limiting the privileges of the researchers in the environment, using virtualization or containerization techniques. Similar to the recommendations for data checks, there is a clear need for tools that can support the TRE team in checking the data and code that researchers wish to bring into the environment. Although there are clear concerns about fully automating this process, developing tools to support these checks can significantly speed up the process and assist with the detection of malicious code.

#### Support for Federated Queries of Data From External Sources

##### Overview

A total of 79% (15/19) of the TREs did not currently support federated queries from external sources, whereas the remaining TREs (4/19, 21%) confirmed that their TRE supported this. A participant described how their TRE can support federated queries via an integration tool on the Health and Social Care Network, using application programming interfaces, XDS.b cross-enterprise document sharing [40], and the Image Exchange Portal (Sectra AB) for imaging.

A federated query entails sending a request to ≥1 external data sources and receiving only the results of that query, as opposed to the usual pattern of the operators copying an entire data set into the TRE for later analysis. This can be more efficient—avoiding the need to prescreen all data on ingress to the TRE and allowing use of live data sources rather than static copies—and allowing easy aggregation of multiple sources; however, it is hard to implement securely and efficiently.

##### Limitations

Federated queries are difficult to support while maintaining effective privacy and security controls, and they are not currently available in most TREs.

##### Recommendations

Federated queries enable federated learning that can train machine learning (ML) algorithms from diverse data sets without exchanging data. Federated learning can be effective in diagnosing uncommon diseases, and it can also reinforce data privacy and security if the process of data being stored and processed is supported by privacy-preserving and cryptographic techniques [41-43]. Furthermore, federated learning complies with data protection regulations including General Data Protection Regulation (GDPR). However, federated learning is vulnerable to different attacks such as inference attacks (eg, membership and reconstruction attacks) [44] and poisoning attacks [45,46], which can violate GDPR. The possibility of some of these attacks can be mitigated by the application of privacy-preserving mechanisms including secure multiparty computation, differential privacy, and encrypted transfer learning methods [47]; however, differencing attacks remain difficult to guard against [48]. Supporting federated queries of data from external sources is a feature of interest for the next-generation TREs.

#### Audit and Workflow Management

##### Overview

Audit and monitoring are key aspects of a TRE. Many participants reported that they use project management tools to automate functions such as Jira [49], which can be customized to record transitions, such as a request being made by a user, review of the data to be exported, and subsequent acceptance or rejection of the export. Most TREs (13/19, 68%) reported that they keep a copy of the exported data.

The level of automation and functionality of auditing differs between TREs. The state of the art includes the following:

1. Real-time alerting on the digital airlock, providing a verbose description of user activity. The reports and alerts generated from this provides the IP address of the user and their username, along with the time, date, file name, file size, and few other supplementary fields.
2. All the activities in the TRE are logged, dashboards are used to support the monitoring of the activities, and reports are automatically generated.
3. If a user attempted to export data that was not permitted, this will be logged. If abnormal patterns are observed, the antimalware software (eg, Sophos plus quest tools [50]) will trigger alerts and log tickets on the system. The technical team and data owner will receive an email alert advising them that abnormal patterns had been detected.

##### Limitations

Many TREs have little or no automation and automated auditing in place, thus limiting the available reporting and operational insights.

##### Recommendations

Incorporating a logging and monitoring system into the TRE is important. This system can include log-in attempts, including username, time and date of access, IP address, type of activity conducted during the session (eg, which tools were used, for how long, and any processes that were running), details of any imports and exports (including file name and file size), and access type (successful or denied). Furthermore, having a real-time alert system can warn the TRE team promptly in case of any malicious attempts and assist in preventing unwanted disclosure and blocking access.

### Safe Computing

Participants were asked whether their TRE uses private (on-premises) or public cloud infrastructure (“Infrastructure as a service,” such as Amazon’s Amazon Web Services or Microsoft’s Azure). In total, 64% (14/22) of the participants reported that their TREs use a private cloud. There were some concerns from these participants that data governance restrictions may make switching to a public cloud difficult. In all, 21% (4/19) of the TREs were already hosted in public clouds, and 10% (2/19) of the participants reported that they aim to switch to a public cloud in the future. Although costs are generally high in public clouds, the extra functionality and flexibility make this an attractive option when possible.

In comparison with local (“on-premise”) servers, storing data, especially sensitive data (eg, health data), in the public cloud can be considered more secure because of its enhanced security measures and the expert advice available from the cloud service providers. However, most TREs currently use on-premise servers, which simplify data governance and data locality issues and provide consistent performance and predictable costs. It was interesting to see that some TREs plan to switch to public cloud in the future, as the public cloud can scale more easily and provide access to next-generation services such as AI or ML, containers, and so on.

### Safe People

The safe people controls are measures and policies to ensure that trusted researchers will use the platform in an appropriate manner.

#### Controls on the People Who Use the TRE

##### Overview

Best practices for ensuring that the researchers accessing the environment are trustworthy and understand the importance of correct use of the TRE include the following: signing legal documents to agree that a researcher will avoid attempting to reidentify any individual, rapid disclosure of any vulnerabilities detected by a researcher, keeping log-in credentials private, and notification to the TRE if a researcher was leaving their institution. A participant reported that financial penalties can be a useful deterrent to misuse.

Moreover, 85% (17/20) of the participants responded that researchers using their TREs are required to complete training. This training typically consists of information governance, GDPR, awareness of issues related to privacy, ethics, security, information security, Medical Research Council training, and statistical disclosure control. Researchers are typically required to complete the training annually or before the beginning of each project. The nature of this training and subsequent contract or terms of use are typically determined by the data owner. For example, government security clearance is requested by the Defence Science and Technology Laboratory for access to their data.

In 79% (15/22) of TREs, researchers sign an agreement not to misuse the environment or the data. This agreement is also signed by a senior member within each organization. A participant stated that if a researcher is a student, a supervisor also needs to sign the agreement. There was a range of penalties applied across the TREs for violating the user agreement, which, in the most extreme form, can result in job loss, disciplinary measures, or, in some cases, compulsory retraining. Project approval is also required by the relevant data controller, and in some cases, the project also has to be signed by an ethics committee. According to a TRE, conditions specified in a nondisclosure agreement or access request form will impose constraints regarding appropriate use of the data and can pass all responsibilities for ensuring that the data were being used correctly on to the sponsoring organization.

##### Recommendations

Training, such as information governance training, is vital to ensure that researchers understand their responsibilities and should be considered by all TREs. Through training, researchers will clearly understand what they are allowed to do with the data. TREs must implement suitable review and management processes to further ensure that researchers are using the TREs appropriately. Best practice approaches to delivering effective training for TRE researchers, which not only support the use of the TRE but also facilitate building shared attitudes and responsibilities in protecting data, are widely available [36,37,51].

#### Controlling Access to the Environment for Trusted Users Only

Overall, 76% (14/22) of the participants stated that access to their TREs is limited to those researchers who are associated with an approved (trusted) organization. Furthermore, 14% (2/14) of these participants stated that access was limited to organizations in the same country as the TRE, as specified by the data custodian. For 7% (1/14) of them, commercial organizations were not allowed to access the TRE under any circumstances. For other TREs that permit commercial organizations to access the environment, the criteria for approving these organizations were generally set higher than those for other organizations (eg, universities). In a TRE, although requests from commercial organizations were considered, they needed a university sponsor or health sponsor to be approved. Another participant responded that commercial customers did not need to be associated with an academic institution. In this case, a review committee determines which projects will be approved for commercial customers.

In a TRE, access is granted only to the users of their own university. In this TRE, an external visitor account will be granted access only if the visitor was sponsored by university staff. In another TRE, researchers can access the environment from a university or NHS-based organization (ie, using white-listed IP addresses). A TRE adopted additional restrictions for the researchers, for example, ensuring that access was permitted only from a safe room or that the device used to access the TRE was a managed device and not a personal device. In this case, these restrictions were set by the data controller.

### Participants’ Recommendations for TRE Enhancements

In all, 27% (6/22) of the participants indicated that they would like improved support for programming capabilities in the environment (eg, Python and R), to advance the analytical capabilities of their TRE and subsequently support large-scale studies. Support for importing data, algorithms, and code to the environment was frequently described as another high-priority feature. However, the licensing of proprietary software tools presents a further limitation regarding the incorporation of software into the environment, because not all licenses cover use within a TRE.

Overall, 9% (2/22) of the participants confirmed that they would like to support federated learning to advance data movement among TREs, where data sets need to be shared and accessible. Support for additional data modalities, such as imaging and genomic data, needs to follow a proper risk assessment, and TREs would have to ensure that they liaise with data custodians regarding the specific risks. It was widely acknowledged by the participants that there were many security challenges around allowing researchers to bring their own data and code into the environment, and until solutions to these challenges have been developed, many TREs will be reluctant to support this.

A total of 23% (5/22) of the participants indicated that they would like to simplify the process for researchers to access data within their TRE in the future. The process and checks required before researchers are granted access to the data were perceived as cumbersome and slow. Sometimes, this administrative process is further delayed owing to backlog of project review requests, committees being slow to make decisions, ethics board approval, and researchers completing relevant training courses and privacy training. Researchers are eager to have access to the TRE and its data promptly; hence, TREs desired to simplify this process. Overall, 36% (8/22) of the participants discussed how they would like to improve the governance processes. A participant stated that all data sets in their TRE were treated as high risk and had to go through the same governance process, even though some data sets were actually low risk. A participant suggested that it will be useful to conduct a national risk-benefit analysis of sharing standardized data sets for research. The participant acknowledged that there was no systematic approach to review data sets to determine if there were certain conditions under which these can be used by researchers without the full governance checks.

Some TREs are considering migrating to a public cloud for improved scalability and flexibility, including GPU access, great on-demand computing power, and reduced management overheads, whereas several TREs have already made this transition.

A TRE is looking to enhance their security through improved logging of activities, such as data copying between machines, and better behavior tracking.

Finally, a participant discussed concerns about intellectual property when the code was developed within the TRE. The participant acknowledged that researchers may have concerns regarding how the code that they develop or test in a TRE can be accessed by the TRE operators. Policies and practices to govern this should be established to protect both parties. Technical solutions to this problem, such as trusted computing and enclave approaches, can also be explored.

## Discussion

This study reviewed the existing controls used by UK and international TREs that participated in our structured interviews. These controls cover a subset of the seven “Safes,” comprising safe people, safe setting, safe computing (an extension of safe setting), safe data, and safe outputs. The features that most need further work for next-generation TREs are the following:

1. Advancing analytical power (high-performance computing clusters and GPUs) available within the environment to support large-scale studies
2. Bringing data, algorithms, or code into the environment and addressing the security challenges arising from this
3. Being able to develop ML and AI algorithms within the TRE and export them
4. Supporting federated queries of data from external sources
5. Supporting additional data modalities such as imaging and genomic data
6. Simplifying the process of accessing data for researchers
7. Scalability

This study analyzed the extent to which TREs can support the import and export of different data types. The process used is largely manual, with some TREs using software to support this process. Finding suitable software to support the automation of the data center process was identified as a key priority for most TREs. Furthermore, the application of ML techniques in TREs can be useful for predicting the malicious use of accessed data by researchers. It was evident that most TREs do not have specific tools to manage and mitigate the potential risk of reidentification, and they rely on analysts’ knowledge and judgment and communication with the data controller.

There is a lack of support for AI and ML development in TREs, and there is a concern that researchers can perform malicious activities owing to the AI and ML structure, for example, exporting sensitive data that can be vulnerable to exposure following attacks against the AI model or overtraining the AI algorithm. The difficulties in detecting these exports were acknowledged as a significant challenge by the participants.

The computing power available to researchers is generally adequate for current needs (observational studies using statistical analysis tools); however, there was a clear desire to ensure that this was scalable to meet researcher’s requirements for analyzing big data and for AI development. Some TREs already appear to be significantly constrained. There is significant variety in the extent of the security measures used to mitigate the risk of unauthorized access, data loss, and misuse by the researcher, and there are some concerns regarding the implications of next-generation capabilities on the security of the TRE and protecting the data. Furthermore, there is a need for advanced information governance for TREs encompassing incoming and outgoing automated data feeds, ad hoc incoming data and algorithms, and ad hoc outgoing data and algorithms. Finding appropriate solutions to meet these needs should be explored in future studies.

Alternative approaches to the remote desktop approach exist, and further exploration of the relative merits of these alternatives will be valuable, particularly in the context of new and evolving types of data.

## Appendix

Multimedia Appendix 1 Interview questions.

## Abbreviations

AI: artificial intelligence
GDPR: General Data Protection Regulation
GPU: graphics processing unit
ML: machine learning
NHS: National Health Service
TRE: Trusted Research Environment
VM: virtual machine
