# Supplementary material for: Next-Generation Capabilities in Trusted Research Environments: Interview Study
Source: J Med Internet Res. 2022 Sep 20;24(9):e33720. doi: 10.2196/33720 (PMC9533202; doi:10.2196/33720)
Supplement: Multimedia Appendix 1 [file jmir_v24i9e33720_app1.docx]

# Appendix A – Interview Questions

1. Background Information:
   1. Name of the organisation that you work for:
   2. Name of the Data Safe Haven (DSH) that you work for:
   3. Job title:
   4. Brief explanation of your role in the DSH:
   5. Can you describe what works well in your DSH?
   6. Can you describe what features you would like to see in place to improve the DSH?

# Safe Outputs

1. Select what types of data a researcher can export from the DSH:

- Aggregate level graphs and tables
- Individual level, anonymised data
- Weights of an AI algorithm
- Weights and code for an AI algorithm
- Software source developed within the DSH
- Software executable developed within the DSH
- Other types of data – please list

a) Do you receive requests to export other data types? If yes please describe.

b) Do you have any plans to enable export of any additional types of data in the future? If yes

please describe.

1. Describe the process you use for checking disclosure control on data to be exported. Please cover
2. what software you use or consider for this purpose
3. are there any issues (eg cost, rate of false positives) that have prevented suitable software being adopted
4. when the data can be exported (e.g. once, daily)
5. how that data can be exported
6. what restrictions do you impose on this process?
7. If software is used for disclosure control (as described in question 4), what checks does this software perform?
8. What manual checks do you use for disclosure control?

a) Have any known breaches or near-miss incidents occurred? (For example, was a spreadsheet

file cleared for export without realising there was additional data stored within the Undo

history?)

b) Are you aware of any potential gaps in your disclosure control checks?( For example, could a

researcher change the colour of text to the same as the background colour of a file to be

exported and this be missed by the manual checks? )

1. What is the minimum number of individual data points allowed within a cell to be exported? For example, a cell count within a table has to be >5 individuals.

# Safe Data

1. As part of strengthening safeguards, which tools and techniques are used in your DSH to manage and reduce the potential risk of re-identification?

1. Digital watermarking is a technology in which identification information is embedded into the data carrier in ways that cannot be easily noticed, and in which the data usage will not be affected. This technology often protects the copyright of multimedia data and protects databases and text files. Digital watermarking can be effectively used to trace disclosure of health data. Do you think watermarking can be used to preserve health data privacy when data is disclosed to researchers via DSH?

- Yes
- No

If yes, have you used this approach on your developed DSH? Describe your approach.

# Safe Setting

- 1. What computing power do you offer a “standard” DSH user (CPUs, GPUs, memory and storage)?
  2. What OS is used on your “standard” build (Windows/Linux/other)?
  3. What environment do you offer if a VM is not used? (For example, Amazon SageMaker for building machine learning pipelines in a web interface)

1. What is the maximum computing power you offer DSH “power” users (CPUs, GPUs, memory and storage)?
2. What are the data security measures that are employed in your DSH to mitigate the risk of the following?
   1. Unauthorized access
   2. Data loss
   3. Misuse by researcher
3. Do you have a standard/base build for the VMs?
   - Yes

- No

If yes, what OS and tools are installed on the VM?


a) Do you implement internal isolation between projects/users/VMs? If yes, how is this managed?

b) What measures do you employ to ensure that researchers cannot execute malicious code in the

environment?

1. What measures do you employ to control any external access to the VM ( e.g. USB, connecting

external drives, connecting to the internet)?

a) What security checks do you employ on the VM?

b) Do you have internal red team/testers that check the security?

1. Do you allow the researchers to have a custom-built environment?

If yes, what security measures do you employ to check the custom VM?

1. Do you allow researchers to modify the environment at a later stage?

If yes, what security measures do you employ to check the modified environment?

1. Are researchers allowed to import data or code (including libraries) into the environment?

If yes, what security measures do you employ at this stage? For examples how do you scan imported software/tools to ensure that they will not compromise the security and integrity of the DSH?

1. Does your DSH support federated queries of data from external sources? If so, please give details.

# Safe Computing (an extension of Safe setting)

1. Is your DSH a private or public cloud? Please provide details.

# Safe People

1. What controls do you put on the people who use the DSH? For example, data governance training, signing a legal document with terms and conditions of use.
2. Does access to the environment have to be via a recognised “trusted” organisation i.e. from a university network within the UK? Please provide details.
3. Can the environment be accessed from anywhere in the world? If not, please provide specifics.

**Evaluating the functionality of the DSH**

Based on your experience with DSH, please indicate the extent to which you agree (or disagree) with the following statements:

| **Statement** | **Strongly**  **agree** | **Agree** | **Neutral** | **Disagree** | **Strongly disagree** |
| --- | --- | --- | --- | --- | --- |
| DSH is a dynamic instrument that can contribute to future developments in the science, technology, and practices of genomic and health-related data sharing. |  |  |  |  |  |
| DSH can serve as a tool for the evaluation of responsible research by research ethics committees and data access committees. |  |  |  |  |  |
| Information security and privacy controls that are applied to DSH can be applied effectively to all operations, services and systems that process sensitive data. |  |  |  |  |  |
| Anonymization of Personally Identifiable Information (PII) or Protected Health Information (PHI) on DSH can preserve data privacy and mitigate the possibility of re-identification and information leakage. |  |  |  |  |  |
| Application of machine learning techniques on DSH can be useful in predicting the malicious use of accessed data by researchers. |  |  |  |  |  |
| Identifiers, recognisable identifiers, and sensitive attributes must be removed completely. |  |  |  |  |  |
| DSH owner can fully ensure that the researcher only uses the data appropriately. |  |  |  |  |  |
| There is no concern that accessing data in the DSH may be damaging to the patient. |  |  |  |  |  |
| Statistical results must not be disclosed in the public domain. |  |  |  |  |  |

# Additional Questions If Time Allows

1. Which of the following are important for you to consider within your DSH?

- Data privacy
- Fine-grained access control
- Efficiency
- Scalability
- Ability to export data
- Ability to export algorithms
- Ability to import existing algorithms from external storage

1. Does your organisation have any policies or principles governing the safe use of data in your DSH?

- Yes
- No

If yes, are these policies or principles influenced by any standards, laws of government

polices?

- Yes
- No

If yes, can you list them?

1. Is your DSH approved / validated? If so by which organisation?
2. In your opinion, which of the following principles do you think is the most important to ensure the responsible sharing of genomic and health-related data?

- Respect Individuals, Families and Communities
- Advance Research and Scientific Knowledge
- Promote Health, Wellbeing and the Fair Distribution of Benefits
- Foster Trust, Integrity and Reciprocity

1. Who is responsible for managing your data security and privacy program?
